# Acute high glucose exposure impairs synaptosomal vesicle release activity

**DOI:** 10.1016/j.brainres.2025.149751

**Published:** 2025-06-01

**Authors:** Nadine Alshakhshir, Lucy He, Liqin Zhao

**Affiliations:** a Department of Pharmacology and Toxicology, School of Pharmacy, Lawrence, KS 66045, USA; b Neuroscience Graduate Program, University of Kansas, Lawrence, KS 66045, USA

**Keywords:** Alzheimer’s disease, Diabetes, Hyperglycemia, Synaptosomes, Synaptic exocytosis, V-ATPase

## Abstract

**Background:**

Increasing evidence indicates an association between Alzheimer’s disease (AD) and diabetes. AD and diabetic brains share a hyperglycemic phenotype, making it a plausible mechanistic link between the two diseases. The vacuolar-type ATPase (V-ATPase) is essential for neurotransmitter concentration in synaptic vesicles and subsequent neuronal transmission. However, its role in AD pathogenesis is unclear.

**Objectives:**

We sought to examine whether acute hyperglycemic exposure would alter synaptic vesicular exocytosis and V-ATPase function in synaptosomes freshly extracted from wildtype mouse brains.

**Methods:**

Synaptic exocytosis was studied by analyzing the synaptosomal release of the fluorescent dye acridine orange (AO) and the neurotransmitter glutamate. Synaptic V-ATPase activity was assessed by measuring V-ATPase assembly using co-immunoprecipitation and V-ATPase-released phosphates.

**Results:**

Acute hyperglycemia reduced synaptic vesicular exocytosis as indicated by attenuated AO and glutamate release. Moreover, acute hyperglycemia reduced synaptic V-ATPase assembly but not V-ATPase-released phosphates.

**Conclusions:**

The present work demonstrates that hyperglycemia can impair synaptic vesicular exocytosis, partially by reducing V-ATPase assembly. We hypothesize that these molecular changes can be a shared mechanistic contributor to synaptic dysfunction in AD and diabetes. Further studies are needed to investigate the impact of chronic hyperglycemia and glycolytic metabolism on synaptic vesicular activity and V-ATPase function in animal models of AD and diabetes.

## 1. Introduction

Alzheimer’s disease (AD) is a progressive neurodegenerative disorder characterized by the abnormal accumulation of amyloid β (Aβ) peptides and hyperphosphorylation of tau protein, leading to the deposition of extracellular amyloid plaques and intracellular neurofibrillary tangles. Other pathological changes include a chronic inflammatory state, bioenergetic deficits, synaptic impairment, neuronal death, and brain atrophy.

There is a growing body of evidence indicating a strong connection between AD and diabetes, whch manifests in multiple aspects such as shared pathological features and increased susceptibility to developing AD among diabetics. Specifically, AD brains display deficits in the insulin signal transduction pathway where they have reduced expression of insulin, insulin growth factors (IGFs), insulin and IGF receptors, and downstream signaling molecules (Candeias et al., 2012; Steen et al., 2005). Diabetic mouse models present AD-like neuropathologies, including reduced brain weight, mitochondrial dysfunction, reduction in synaptic and autophagy-related proteins (Carvalho et al., 2015), impaired synaptic plasticity (Liu et al., 2015), cognitive deficits (Ma et al., 2017; Malone et al., 2008), hippocampal damage and apoptosis, decreased lysosomal markers, and increased Aβ1–42 expression (Ma et al., 2017). Moreover, studies have shown that animal models with both AD and diabetes have exacerbated brain pathologies compared to models with AD or diabetes alone (Guo et al., 2016; Wang et al., 2015). Furthermore, diabetes increases the risk of cognitive impairment and triggers the onset of AD. In two independent longitudinal prospective studies, diabetes increased AD risk by 1.45- and 1.94-fold (Ohara et al., 2011; Wang et al., 2012). AD patients are 1.9 times more likely to be diabetic than non-AD individuals (Janson et al., 2004). Diabetes and hyperglycemia are associated with aggravated cognitive impairment post-stroke (Assayag et al., 2017; Levine et al., 2023). Of particular note, the relative risk of developing AD increases from 1.7 to 5.5 in carriers of the APOE ε4 genotype, the leading genetic risk factor of AD, if they also have diabetes (Peila et al., 2002). These findings underscore the close association between AD and diabetes; however, the underlying mechanisms remain poorly understood.

One significant pathological change observed in AD and diabetes is the dysregulation of glucose metabolism. In diabetes, reduced levels or signaling activity of insulin lead to elevated blood glucose levels, accompanied by increased brain glucose levels (Jacob et al., 2002; Seaquist et al., 2005). AD brains have been shown to exhibit glucose hypometabolism (Minoshima et al., 1997; Mosconi, 2005). Additionally, our study has revealed that APOE ε4 brains have inferior glucose metabolism compared to APOE ε2 and APOE ε3 brains. APOE ε4 brains demonstrated impaired glycolytic activity and mitochondrial respiration and were characterized by the lowest expression profile and activity of the rate-limiting glycolytic enzyme, hexokinase (Wu et al., 2018). Brain hyperglycemia has recently been identified as a new and robust phenotype in AD brains. Elevated glucose levels were observed in AD-sensitive cortical brain regions and positively correlated with AD pathology (An et al., 2018). Taken together, it can be concluded that brain hyperglycemia is a shared phenotype between AD and diabetes, which can potentially be a mechanistic pathological link between the two diseases.

Vacuolar ATPase (V-ATPase) is an ATP-dependent proton pump composed of two multi-subunit domains: a transmembrane V0 domain and a peripheral V1 domain. V-ATPase hydrolyzes ATP and utilizes the produced energy to pump protons across various cellular and subcellular membranes. This activity generates proton electrochemical gradients across biological membranes, thus serving many physiological roles (Hayek et al., 2019; Marshansky & Futai, 2008). Reversible disassembly of V-ATPase is an essential regulatory mechanism of V-ATPase function, during which V0 and V1 domains reversibly disassemble, halting V-ATPase activity (Marshansky & Futai, 2008; Sumner et al., 1995). The two domains can reassemble to resume the V-ATPase function (Kane, 2000, 2012). Multiple studies have shown that glucose levels regulate V-ATPase assembly and activity (Chan & Parra, 2014; Hayek et al., 2019; Kane, 1995; Kohio & Adamson, 2013; Lu et al., 2004, 2007; McGuire & Forgac, 2018; Nakamura, 2004; Sautin et al., 2005; Seol et al., 2001; Smardon et al., 2002). However, these studies mainly focused on the effect of glucose deficiency, whereas the impact of hyperglycemia on V-ATPase remains underexplored.

V-ATPase dysregulation has been shown to be involved in AD pathogenesis, as demonstrated in studies that employed mutations or knockdowns of V-ATPase subunits or V-ATPase inhibitors (Dubos et al., 2015; Lee et al., 2015; Williamson et al., 2010; Williamson and Hie-singer, 2010). Previous work from our lab showed that the ATP6V1B2 subunit of V-ATPase, concentrated in synaptic vesicles where it is essential for neurotransmitter storage and release, is more abundantly expressed in APOE2 brains compared to APOE3 and APOE4 brains. The expression of ATP6V1B2 was also found to be significantly reduced with aging and AD (Woody et al., 2016), further suggesting a potential role of synaptic vesicular V-ATPase dysregulation in AD.

In light of the prominent features of brain hyperglycemia and the dysregulation of V-ATPase in AD, we sought to examine the impact of hyperglycemia on synaptic vesicular exocytosis and V-ATPase function. The results demonstrate that acute exposure to supraphysiological glucose levels, comparable to the levels in diabetic brains, impairs synaptic vesicular release activity and reduces V-ATPase assembly but does not appear to affect V-ATPase-mediated phosphate release.

## 2. Materials and Methods

### 2.1. Materials

Syn-PER^™^ Synaptic Protein Extraction Reagent (Cat #87793), T-PER^™^ Tissue Protein Extraction Reagent (Cat #78510), Halt^™^ Protease and Phosphatase Inhibitor Cocktail 100X (Cat #78446), Pierce^™^ BCA Protein Assay Kit (Cat #23225), N-PER^™^ Neuronal Protein Extraction Reagent (87792), and Co-IP kit (26149) were purchased from Thermo Fisher. ATPase/GTPase activity assay kit (MAK113), β-Nicotinamide Adenine Dinucleotide Phosphate Hydrate (N5755), L-Glutamic acid dehydrogenase from bovine liver (G2501), Adenosine Triphosphate Disodium Salt Hydrate (A2383), Sodium Azide (S2002), Sodium Vanadate (72060), β-Mercaptoethanol (M6250), and Bafilomycin A1 (B1793) were purchased from Sigma. Acridine Orange (14338) was purchased from Cayman Chemicals. 2x Laemmli Buffer (161–0737), Immun-Blot^®^ PVDF membranes (1620177), and Clarity^™^ Western ECL Substrate (170–5061) were purchased from Bio-Rad.

Antibodies: V-ATPase V1 D (Cat #sc-390384), V-ATPase V1 B2 (sc-166045), Normal mouse IgG (sc-2025), and GAPDH (sc-32233) were purchased from Santa Cruz. Anti-ATP6V0D1 (ab202899) was purchased from Abcam. Synaptophysin (5461), PSD 95 (3450), NMDAR2 (4212), and Nuclear Lamin a/c (4777) were purchased from Cell Signaling. VAMP2 (AD1-VAS-SV006-D) was purchased from Enzo. Horseradish Peroxidase conjugated Beta-actin (664804) was purchased from BioLegend.

### 2.2. Animals

The animal work was approved by the Institutional Animal Care and Use Committee at the University of Kansas and followed NIH guidelines for the care and use of laboratory animals. The animals used in experiments included both female and male C57BL/6J mice (2–13 months). They were housed under controlled conditions of temperature, humidity, and a standard 12-h/12-h light–dark cycle with water and food available ad libitum. n = 4–6 animals per experiment. Animals were euthanized, brains were collected, and cortical tissues were processed immediately for downstream applications.

### 2.3. Synaptosome preparation

**Fig. 1.**
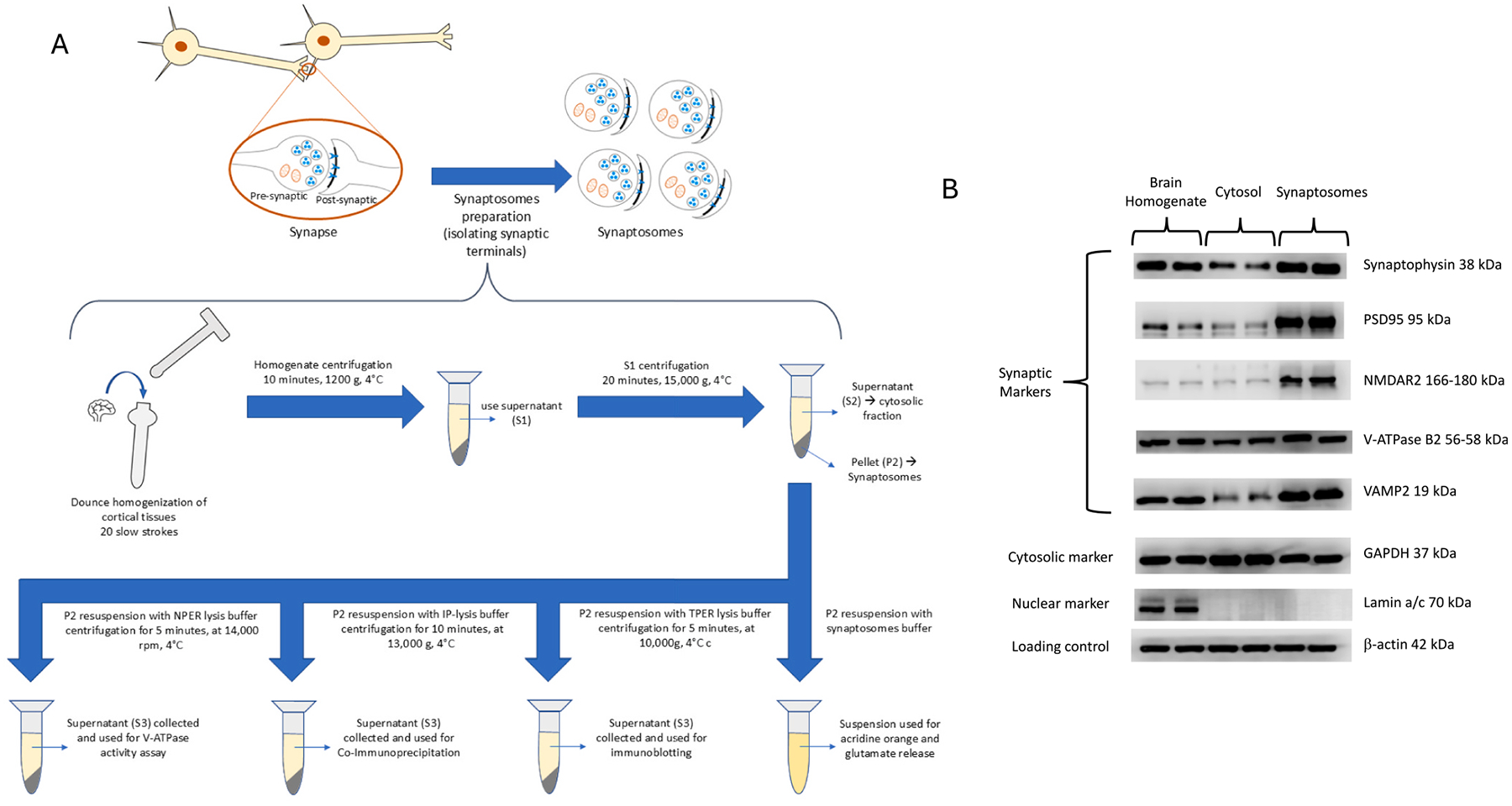
**(A)** Summary of the method used for synaptosome preparation and downstream applications. (**B)** Characterization of the synaptosome fraction shows enrichment of synaptic protein markers compared to the cytosolic fraction and whole brain homogenate, indicating an efficient isolation of synaptosomes.

Synaptosome samples were prepared as per instructions in the Syn-PER^™^ Synaptic Protein Extraction Reagent user guide (# 87793) with some modifications. 200 mg of cortical brain tissues were minced gently and then homogenized with 2 mL Syn-PER reagent and 20 μL of Halt Protease and Phosphatase Inhibitor cocktail (100x). Dounce homogenization was performed (20 slow strokes). The homogenate was centrifuged for 10 min, 1200 g, 4 °C. The supernatant was collected and centrifuged for 20 min at 15,000 g, 4 °C. The produced supernatant (cytosolic fraction) was saved for synaptosome characterization. The pellet (synaptosomes) was resuspended with different buffers in different experiments illustrated in Fig. 1A. The prepared synaptosomes fraction was characterized by comparing the levels of synaptic protein markers (Synaptophysin, PSD95, NMDAR2, V-ATPase V1 B2, and VAMP2), a cytosolic marker (GAPDH), and a nuclear marker (nuclear Lamin a/c) in the synaptosome fraction relative to the cytosolic fraction and the whole brain homogenate. Immunoblotting was used for analysis. Dilutions of the used primary antibodies are as follows: GAPDH (1:5,000), Synaptophysin (1:1,000), PSD95 (1:1,000), Lamin a/c (1:2,000), V-ATPase V1 B2 (1:1,000), VAMP2 (1:1,000), NMDAR2 (1:1,000).

### 2.4. Acridine orange release

Light exposure was kept to a minimal level during acridine orange solution preparation and experiments, the dye solutions were prepared fresh each time the experiment was conducted. Experiments were performed as described in (Hrynevich et al., 2016) with some modifications. Synaptosomal pellets were resuspended with synaptosome buffer to produce the synaptosome stock from which samples were prepared. The synaptosomes buffer contained 132 mM NaCl, 5 mM KCl, 1.3 mM MgCl_2_, 1.2 mM NaH_2_PO_4_, and 15 mM HEPES. The buffer pH was adjusted to 7.4. Samples were prepared from synaptosome stock at 2.5- or 10-mM final glucose concentration for normoglycemia and hyperglycemia (rationale for glucose levels explained in Section 3.1), respectively, and 0.5 mg/mL protein concentration. CaCl_2_ and dye (final concentrations 2 mM and 5 μM, respectively) were added to samples just before incubation. Triplicates of 100 μL sample were loaded into black 96-well plates and incubated for 10 min at 37 °C. After incubation, kinetic fluorescence measurement was performed using Molecular Devices plate reader SpectraMax iD3 at excitation/emission wavelengths 490/530 and the plate reader chamber temperature at 37 °C. Baseline fluorescence was recorded for 2–3 min, and KCl was then injected into the wells to a final concentration of 60 mM. After which, plate shaking was performed, and fluorescence reading resumed. Blank samples contained dye without synaptosomes. The increase in fluorescence reading after KCl injection relative to baseline was used for analysis (acridine orange release = highest fluorescence value in the first minute after KCl injection – average baseline fluorescence).

### 2.5. Glutamate release

Experiments were performed as described in (Nicholls et al., 1987) with some modifications. Synaptosomal pellets were resuspended with the synaptosome buffer to produce the synaptosome stock as done in acridine orange experiments. Samples were prepared from synaptosome stock in special optical glass fluorescence cells, with 2.5- or 10-mM final glucose concentration, 0.2 mg/mL protein concentration, and 16 μM bovine serum albumin (BSA). CaCl_2_ and Nicotinamide Adenine Dinucleotide Phosphate NADP (final concentration 1.3 mM and 1 mM, respectively) were added to samples 4 min after the incubation started. After completion of the incubation (10 min at 37 °C), kinetic fluorescence reading was performed using a Perkin Elmer FL 6500 Spectrofluorometer using an accessory that allows temperature control (calibrated to 37 °C on every experiment day) with constant mixing using magnetic stirrers. Reading was recorded for one minute, after which L-Glutamic Acid Dehydrogenase (L-GDH) enzyme (5.6 units/mL) was added to the sample, and 1.5–2 min later, KCl was injected at a final concentration of 120 mM. The released glutamate would undergo oxidative deamination in the presence of the L-GDH enzyme and NADP, producing α-ketoglutarate, ammonia, and NADPH. NADPH fluorescence was monitored at excitation/emission wavelengths 340/460 nm as an indicator of released glutamate. The rate of NADPH signal change compared to the baseline was used for analysis (glutamate release = (rate of NADPH signal change 10 s after KCl injection) – (rate of NADPH signal change 10 s before KCl injection)). As calcium is essential for exocytosis, synaptosome preparations without CaCl_2_ were used as negative controls.

### 2.6. Co-Immunoprecipitation

Synaptosomal pellets were resuspended with IP-Lysis buffer provided with the Co-IP kit, followed by incubation on ice for 20 min, then centrifugation for 10 min, 13,000 g, 4 °C. The supernatant was collected and used as a sample stock. 50 μL of sample stock was diluted with IP-Lysis buffer to prepare 250 μL samples with final glucose concentrations of 2.5 or 10 mM. Samples were incubated at 37 °C for 10 min, then 200 μL (150 μg protein) was transferred to columns for pre-clearing with Pierce Control Agarose Resin (Co-IP kit), which was performed for 1 h at 4 °C on a rotator. Pre-cleared samples were then transferred to columns with an immobilized antibody against the V1 D subunit of V-ATPase in order to perform V-ATPase complex capture, or immobilized with normal mouse IgG as the negative control. V-ATPase capture was performed overnight at 4 °C on the rotator. The following day, the samples were eluted with elution buffer (from the Co-IP kit) and diluted with 2x Laemmli buffer with β-mercaptoethanol, then heated for 5 min at 95 °C. Equal amounts of protein were separated by sodium dodecyl sulfate–polyacrylamide gel electrophoresis (SDS-PAGE) using 10 % acrylamide gels, then transferred to 0.2 μM PVDF membranes for one hour at 100 V. Membranes were then cut at 50 kDa and two membranes were produced, the membrane of < 50 kDa was used to detect the V0 domain using an Anti-ATP6V0D1 antibody, and the membrane of > 50 kDa was used to detect the V1 domain using V1 B2 antibody which binds to a V1 subunit that is distinct from the one used for V-ATPase capture (V1 D). Membranes were then blocked for at least one hour at RT with 5 % non-fat milk in TBST and then incubated with Anti-ATP6V0D1 antibody (1:5000 dilution) or V1 B2 antibody (1:1000 dilution) overnight at 4 °C in 3 % non-fat milk in TBS. The following day, the membranes were incubated with horseradish peroxidase-conjugated secondary antibodies and then incubated with ECL substrate for 5 min. The membranes were then scanned using a C-Digit Blot Scanner (LI-COR, Lincoln, NE). The ratio of protein band intensities (V0/V1) was used as an indicator of V-ATPase assembly.

### 2.7. Immunoblotting

Samples with equal amounts of protein were prepared and diluted with 2x Laemmli buffer with β-mercaptoethanol, then heated for 5 min at 95 °C. Equal amounts of sample were separated by SDS-PAGE using 10 % acrylamide gels, then transferred to 0.2 μM PVDF membranes for one hour at 100 V. Membranes were then blocked for at least one hour at RT with 5 % non-fat milk in TBST and then incubated with primary antibodies overnight at 4 °C in 3 % non-fat milk in TBS. The following day, the membranes were incubated with horseradish peroxidase-conjugated secondary antibodies and then incubated with ECL substrate for 5 min. The membranes were scanned using a C-Digit Blot Scanner (LI-COR, Lincoln, NE). For loading control, membranes were stripped for 8 min, then blocked for 1 h at RT with 5 % non-fat milk in TBST, then incubated with HRP-conjugated β-actin (1:20,000 dilution) in 3 % non-fat milk in TBS for one hour at RT. The membrane was incubated with ECL substrate for 5 min and then scanned.

V-ATPase expression control using immunoblotting: Synaptosomal pellets were resuspended with TPER, left on ice for 20 min, then centrifuged for 5 min at 10,000 g, 4 °C. The supernatant was collected and used as a sample stock. Equal amounts of the sample stock were used to prepare samples with final glucose concentrations of 2.5 or 10 mM. Samples were incubated at 37 °C for 10 min, then immunoblotting was performed to test if V-ATPase expression is altered under hyperglycemia. After the transfer step in Immunoblotting, membranes were cut at 50 kDa, and two membranes were produced; the membrane of < 50 kDa was used to detect the V0 domain using the Anti-ATP6V0D1 antibody, and the membrane of > 50 kDa was used to detect the V1 domain using the V1 B2 antibody. Primary antibody dilutions: Anti-ATP6V0D1 antibody (1:5000 dilution) and V1 B2 antibody (1:1000 dilution).

### 2.8. V-ATPase activity assay

Similar to other types of ATPases, V-ATPase hydrolyzes ATP to produce a mole of ADP and inorganic phosphate (Pi). This assay measured phosphates produced by V-ATPase as an indicator of V-ATPase activity. P- and F-Type ATPases were inhibited to ensure the measured phosphate levels were specifically originating from V-ATPase. This assay was performed as per the ATPase/GTPase activity assay kit instructions and the method described in (Pertl-Obermeyer & Obermeyer, 2013) with some modifications.

Synaptosomal pellets were resuspended with NPER, left on ice for 20 min, then centrifuged for 5 min at 14,000 rpm, 4 °C. The supernatant was collected and used as a sample stock. The sample stock was diluted using the assay buffer to produce final samples that have 1 μg protein/40 μL. Samples with either 2.5 mM or 10 mM glucose were incubated at 37 °C for 10 min, after which they were incubated with ATPase inhibitors for 30 min at RT on a rotator. Each glucose concentration had two samples; one was incubated with inhibitors for the three types of ATPases (100 nM Bafilomycin A1 for V-ATPase, 1 mM Sodium Azide for F-ATPase, and 200 μM Sodium ortho Vanadate for P-ATPase), while the second sample replaced V-ATPase blocker bafilomycin A1 with the vehicle (methanol).

After inhibiting ATPases, samples were incubated with 1 mM ATP to start the reaction and incubated for one hour at RT on a rotator, blanks received assay buffer instead of ATP. After which, 40 μL samples were loaded into clear 96-well plates (1 μg protein/well), and 200 μL of malachite green reagent (from the assay kit) was added to samples and blanks in order to terminate the reaction and generate a colorimetric free inorganic phosphate product, samples were incubated with reagent for one hour at RT then absorbance was measured at 620 nm. V-ATPase activity was calculated by subtracting the blank absorbance from samples absorbance values, which was followed by subtracting the values of Bafilomycin A1 samples from ones that contained all three inhibitors (V-ATPase activity = V-ATPase produced phosphates (Pi) = (Pi_All ATPases inhibitors_-Pi_blank_) (Pi_ATPases inhibitors w/o bafilomycin_-Pi_blank_)). A phosphate calibration curve was constructed on every experiment to ensure that the readings were within the dynamic range.

### 2.9. Statistical analysis

RStudio (RStudio software, Boston, MA, USA) was used to perform statistical analysis. Student’s *t*-test was used for quantitative analysis. To minimize inter-experimental variability, values obtained from each experiment were normalized to the normoglycemia group (2.5 mM). Data was presented in box plots; the whiskers extend from the 5th to 95th percentiles. P-values lower than 0.05 were considered statistically significant.

## 3. Results

### 3.1. Brain normo- and hyperglycemic levels

When deciding the glucose levels to use in experiments, we aimed to choose levels that would be physiologically relevant to the brain. We observed that some studies exposed brain tissues to glucose levels usually considered normal or high in the blood. Since blood glucose levels do not necessarily reflect the glucose concentrations in the brain, we reviewed studies that specifically measured brain glucose levels and their relation to blood glucose under normal and hyperglycemic conditions. These studies administered dextrose peripherally while recording blood and brain glucose changes.

Primarily, we observed that brain glucose levels are not equal to blood glucose levels. Brain glucose levels are lower than blood levels and are at a 0.20–0.34 ratio of blood glucose. Brain glucose levels increase linearly with blood glucose in the tested ranges (Gruetter et al., 1998; Jacob et al., 2002; Seaquist et al., 2005; Silver & Erecinska, 1994). In one study, 18 healthy participants received dextrose infusions to reach blood glucose levels between 4–30 mM while monitoring brain levels. Results showed a linear correlation between the levels of the two compartments, where the brain levels ranged between 1–9 mM (Gruetter et al., 1998). Consistent results were found in a study performed on Wistar rats; the ratio of the brain to blood glucose under normo- and hyperglycemia was 0.31 and 0.29, respectively (Silver & Erecinska, 1994). Moreover, inducing peripheral hyperglycemia elevates brain glucose at a similar brain-blood ratio in both healthy participants and participants with chronic diabetic hyperglycemia, which is observed in humans and rats (Jacob et al., 2002; Seaquist et al., 2005). In our experiments, we wanted to expose synaptosome preparations to glucose levels that closely resemble the normo- and hyperglycemic values in the brain. Thus, we chose 2.5 mM and 10 mM glucose to represent the brain’s normo- and hyperglycemic conditions, respectively.

### 3.2. Hyperglycemia reduced synaptosomal acridine orange release

To validate our synaptosome model, we compared the levels of synaptic markers in synaptosome preparations versus the whole brain homogenate and the cytosolic fraction. Western blotting showed enrichment of synaptic protein markers in the synaptosomes fraction, including synaptophysin, PSD95, NMDAR2, V-ATPase B2, and VAMP2. In contrast, the cytosolic marker GAPDH was most abundant in the cytosolic fraction. While the nuclear marker (Lamin a/c) appeared in the brain homogenate, it was not present in synaptosomes or the cytosolic fraction (Fig. 1B). These subcellular profiles validate our synaptosome preparation.

**Fig. 2.**
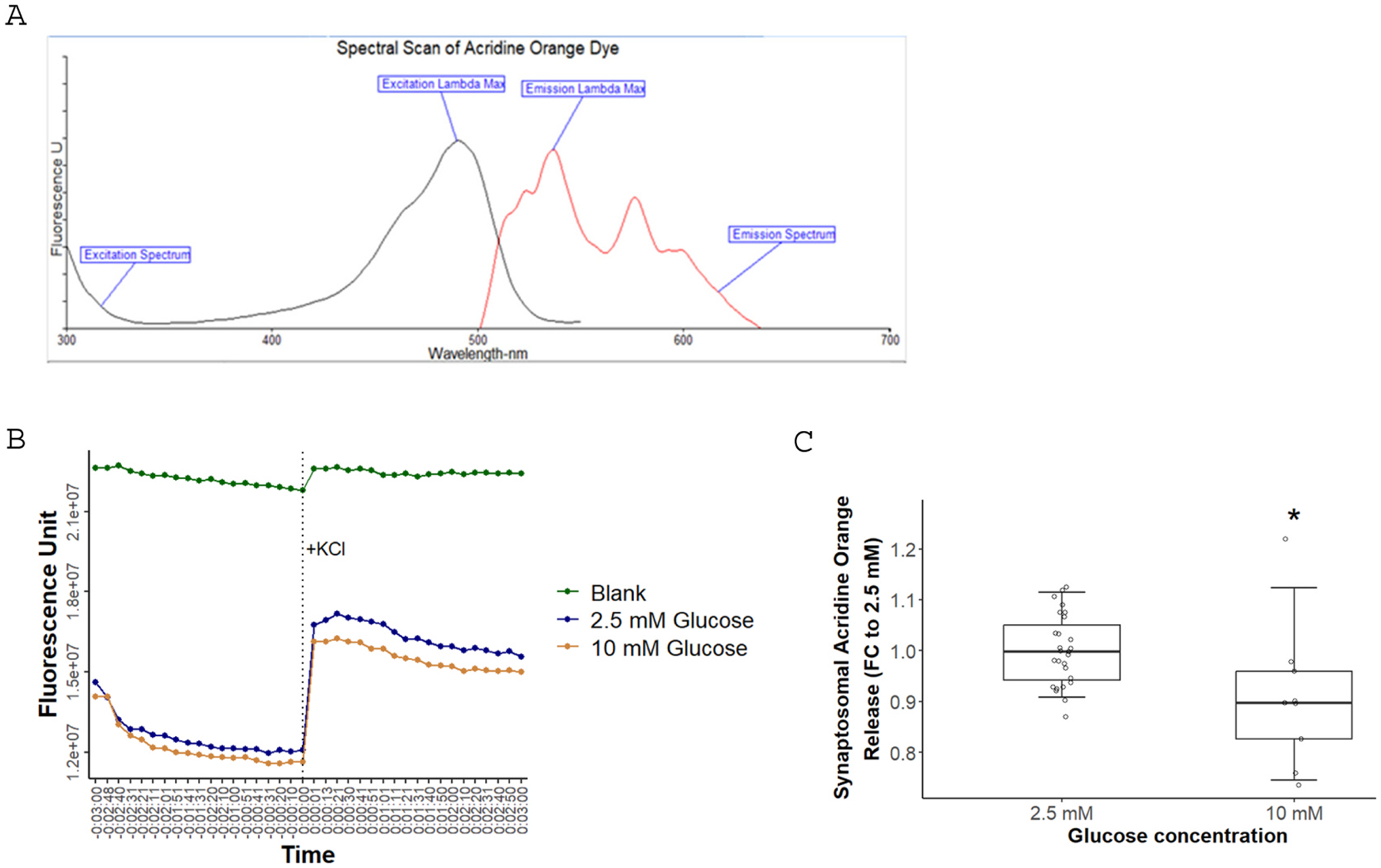
Effect of hyperglycemia on synaptosomal Acridine Orange release. (**A)** Spectral scan of Acridine Orange dye shows the excitation/emission λmax at 490/530 nm. (**B)** A representative plot of the Acridine Orange release experiment. (**C)** Acute hyperglycemia reduced Acridine Orange release by 10 %. A two-sample *t*-test was used for statistical analysis. Experiments were conducted in replicates on 6 female and male mouse brains (n = 6). *: p-value = 0.01395.

Acridine orange is a fluorescent dye, and it is validated and commonly used for studying synaptosomal vesicular release (Hrynevich et al., 2015, 2016; Melnik et al., 2001; Waseem et al., 2006; Zoccarato et al., 1999). Being a weak base, when incubated with synaptosomes, it gets protonated and accumulates in the acidic synaptic vesicles, which leads to aggregation and consequently fluorescence quenching (Amado et al., 2016). Stimulation of synaptic exocytosis through depolarization releases the dye from synaptic vesicles into solution. This results in dye deaggregation and an increase in fluorescence, making it a suitable indicator of synaptosomal release. We performed a spectral scan for the dye using a Perkin Elmer Spectrofluorometer (FL 6500) to determine the λmax of excitation/emission. The spectral scan showed excitation/emission peaks at 490/530 nm (Fig. 2A), which were adopted for experiments.

Synaptosomes were incubated under acute hyperglycemia or normoglycemia, and acridine orange release was performed immediately after the incubation. In order to stimulate synaptic exocytosis, 60 mM KCl was injected into the samples. Injection of high potassium stimulates exocytosis due to the shift of the potassium gradient from being higher intracellularly to higher extracellularly. This decreases the hyperpolarizing outward flow of potassium, triggering an action potential and synaptic release. Fig. 2B shows a representative plot of acridine orange release. The blank sample contained the dye in the buffer without synaptosomes; the blank’s fluorescence signal was higher than the synaptosomes, indicating dye accumulation in synaptic vesicles and fluorescence quenching. The fluorescence increases after exocytosis stimulation indicated the release of dye particles from synaptosomes. The rise of acridine orange fluorescence from baseline after KCl stimulation was reduced by 10 % under hyperglycemia (10 mM glucose) compared to normoglycemia (2.5 mM glucose) (Fig. 2C), suggesting impairment of synaptic exocytosis.

### 3.3. Hyperglycemia reduced synaptosomal glutamate release

**Fig. 3.**
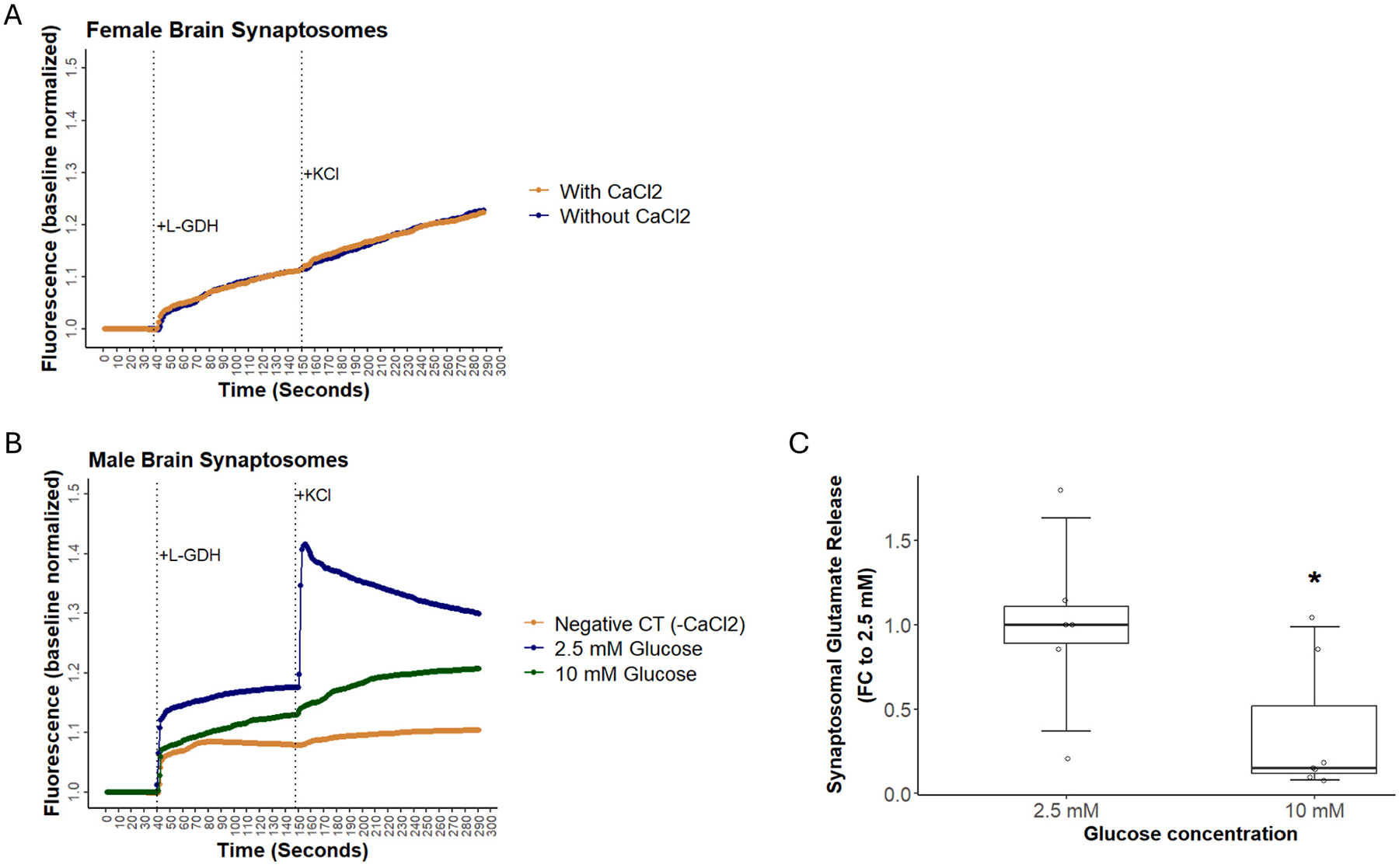
Effect of hyperglycemia on synaptosome glutamate release. **(A)** A representative reading of the synaptosome glutamate release from a female mouse. Female brain synaptosomes showed minimal response to exocytosis, similar to negative controls lacking calcium in the bath. (**B)** A representative reading of the synaptosome glutamate release experiment from male brain synaptosomes. **C)** Acute hyperglycemia reduced male brain synaptosomal glutamate release by 48 %. A two-sample *t*-test was used for statistical analysis. Experiments were conducted in replicates on 4 male mice brains (n = 4). *: p-value = 0.0292.

The effect of hyperglycemia on synaptosomal release was further evaluated using a more physiological approach. This was done by testing the impact of hyperglycemia on the release of the neurotransmitter glutamate. Synaptosomes were incubated under acute hyperglycemia or normoglycemia, and glutamate release was determined immediately after the incubation. L-GDH was added to samples after 40 s of initial reading. To stimulate synaptic exocytosis, 120 mM KCl was added to the samples 1.5 min after enzyme addition. L-GDH performs oxidative deamination on released glutamate, producing NADPH whose fluorescence is measured as an indicator of glutamate release. As calcium is essential for synaptic exocytosis, a negative control sample was used where calcium was not included in the buffer. This experiment showed minimal response in female mice; as shown in Fig. 3A, the reaction to exocytosis in synaptosomes from female brains was similar to negative control without calcium. Thus, data were not analyzed for female mice. Fig. 3B shows a representative reading for a synaptosomal glutamate release experiment from male brain synaptosomes. The negative control shows minimal fluorescence changes after exocytosis stimulation compared to synaptosomes incubated with calcium. Hyperglycemia (10 mM glucose) led to a 48 % reduction in the rate of fluorescence increase upon exocytosis stimulation compared to normoglycemia (2.5 mM glucose) (Fig. 3C). These data are consistent with those obtained from the acridine orange release experiments, further supporting that hyperglycemia may impair synaptic exocytosis.

### 3.4. Hyperglycemia reduced the V0/V1 assembly of V-ATPase

**Fig. 4.**
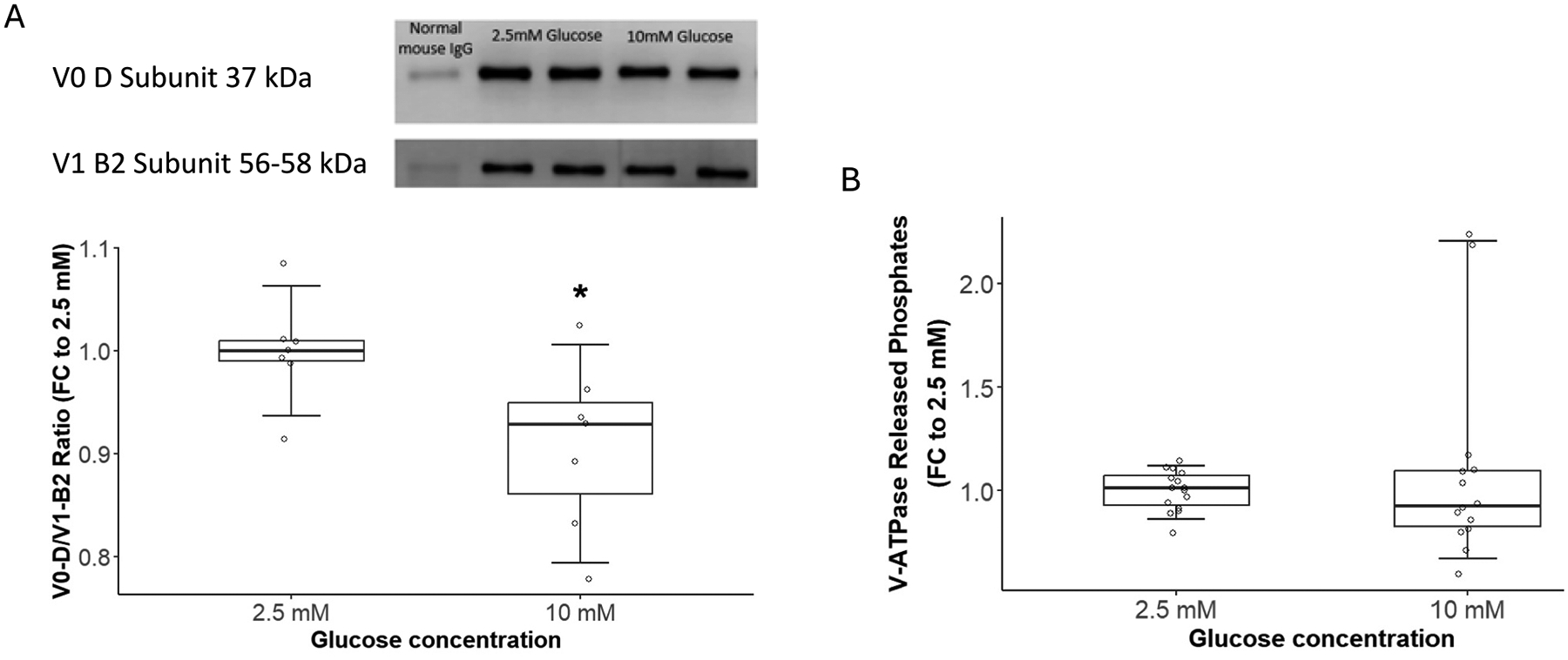
Effect of hyperglycemia on V-ATPase assembly and phosphate release. **(A)** Upper panel: A representative western blot for Co-IP experiments. The V-ATPase complex was pulled down using an antibody against the V1 D subunit. Then, V1 and V0 domain levels were analyzed in a western blot using antibodies against V1 B2 and V0 D subunits. The ratio of (V0-D/V1-B2) band intensities was used as an indicator of V-ATPase assembly. Lower panel: Acute hyperglycemia reduced V0/V1 assembly by 9 %. A two-sample *t*-test was used for statistical analysis. Experiments were conducted in replicates on 4 female and male mouse brains (n = 4). *: p-value = 0.0255. (**B)** Acute hyperglycemia did not influence V-ATPase-released phosphates. A two-sample *t*-test was performed for statistical analysis. Experiments were conducted in replicates on 5 female and male brains (n = 5). p-value = 0.4612.

Reversible disassembly of V-ATPase is a major cellular regulatory mechanism of V-ATPase activity. Thus, we tested the effect of hyperglycemia on V-ATPase assembly using Co-IP. The V1 domain was captured using an antibody against the V1-D subunit of the V1 domain, and antibodies against V1 B2 and V0 D subunits were utilized for subsequent western blotting. Hyperglycemia led to a 9 % reduction in V0/V1 band intensities compared to normoglycemic conditions, indicating reduced V-ATPase assembly under hyperglycemia. A representative blot is shown in Fig. 4A. Statistical analysis showed a significant reduction in V-ATPase assembly under hyperglycemic conditions (Fig. 4A). Since samples were incubated with different glucose concentrations for 10 min, it is unlikely that the expression levels of V-ATPase V1 or V0 domains were altered in this timeframe. We accounted for this factor by conducting Western blot experiments to test if acute hyperglycemia affects V1 or V0 expression. The results showed no effect; hence, the observed reduction in V0/V1 ratio under acute hyperglycemia is unrelated to alterations in V1 or V0 expression.

### 3.5. Hyperglycemia did not affect V-ATPase-produced phosphates

V-ATPase hydrolyzes ATP to generate energy to pump protons across membranes, a process that is accompanied by the release of inorganic phosphates. After we observed a decrease in V-ATPase assembly under hyperglycemia, we tested the effect of hyperglycemia on V-ATPase-specific phosphate release as an indicator of its activity. The results indicate a non-significant effect of hyperglycemia on V-ATPase-released phosphates (Fig. 4B).

## 4. Discussion

The main objective of this study was to investigate the effects of brain hyperglycemia on synaptic vesicular exocytosis and V-ATPase assembly and activity in synaptosomes prepared from wildtype mouse brains. Dysregulation of V-ATPase, particularly lysosomal V-ATPase, has been implicated in AD (Bagh et al., 2017; Colacurcio & Nixon, 2016; Song et al., 2020; Zhao et al., 2018). V-ATPases of synaptic vesicles are essential for neurotransmitter loading (Moriyama et al., 1992) and thus are crucial for synaptic transmission. However, their dysregulation and possible involvement in AD pathogenesis have not been well explored. Brain hyperglycemia has recently been unveiled as a prominent feature of AD brains (An et al., 2018), which can be a possible pathological link with diabetes. The work done in this study was designed to test the hypothesis that brain hyperglycemic conditions impair synaptic transmission through a mechanism involving decreased synaptic vesicular V-ATPase function.

The results showed that acute exposure to glucose at a level comparable to what is found in diabetic brains diminished synaptic vesicular exocytosis and reduced the assembly of the V0/V1 domains of V-ATPase. However, no difference was detected in the number of phosphates released from V-ATPase-mediated ATP hydrolysis. Overall, the results indicate that acute hyperglycemia can impair synaptic exocytosis, which may partly result from reduced V-ATPase assembly. Considering the complexity of the synaptic release process, the observed synaptic impairment could have occurred through a variety of mechanisms.

Synaptic impairment is a main pathological hallmark of AD. The observation on hyperglycemic reduction of synaptic release is consistent with available evidence that shows synaptic dysfunction in diabetic animal models. For instance, diabetic animals exhibited decreased signaling at sympathetic ganglia (Campanucci et al., 2010), impaired axonal transport in olfactory neurons (Sharma et al., 2010), and synaptic plasticity deficits (Biessels et al., 1996; Huang et al., 2015; Li et al., 2002; Martín et al., 2012). Several underlying mechanisms were investigated in these studies, including increased oxidative stress (Campanucci et al., 2010; Sharma et al., 2010), mitochondrial impairment, and reduced ATP levels (Huang et al., 2015). Hyperglycemia has also been shown to be associated with Na^+^/K^+^ ATPase impairment (Torlińska et al., 2006), which might result in disruption of resting membrane potential and action potential physiology, ultimately affecting synaptic transmission. In addition to synaptic dysfunction, diabetes and hyperglycemia are associated with deficits in brain energy production, which might lead to a general disruption of brain activities under hyperglycemic conditions. For example, there is an association with deficits in mitochondrial function (Moreira et al., 2003, 2007; Ortiz et al., 2013; Raza et al., 2015) and autophagic and lysosomal activities (Li et al., 2017; Ma et al., 2017; Sims-Robinson et al., 2016).

The impaired synaptic release could have resulted from the reduced accumulation of neurotransmitters within synaptic vesicles, leading to an overall reduction in neurotransmission. Alternatively, it could be due to reduced synaptic vesicle fusion with presynaptic membranes or a combination of factors. As mentioned earlier, the V-ATPase of synaptic vesicles plays a crucial role in synaptic transmission, as it is essential for loading neurotransmitters into synaptic vesicles. Additionally, the V0 domain of V-ATPase has an important role in synaptic vesicle fusion (Morel et al., 2001; Peters et al., 2001), providing another dimension for the involvement of V-ATPase in synaptic transmission. SNARE proteins (soluble N-ethylmaleimide-sensitive factor activating protein receptors) are a family of proteins involved in vesicle fusion. They are expressed in presynaptic membranes where they bind and form a complex that mediates the vesicle fusion process (Chen & Scheller, 2001). The V0 domain of V-ATPase facilitates vesicle fusion by interacting with SNARE proteins (Morel et al., 2003). There is a possibility that changes in V-ATPase assembly may affect the role of V-ATPase in this process.

AD is increasingly associated with glucose metabolic dysregulation in the brain. For instance, ApoE ε4-expressing cells were revealed to have weakened glycolytic activity and mitochondrial function in comparison with the robust ApoE ε2 bioenergetic profile (Orr et al., 2019; Wu et al., 2018; Zhang et al., 2023). Impaired glycolytic activity is associated with greater AD pathology (An et al., 2018; Vlassenko et al., 2018). AD brains suffer from glucose hypometabolism early on in the disease. Hypometabolism in AD is associated with more severe symptoms, and it can predict AD development and progression (An et al., 2018; Kato et al., 2016; Minoshima et al., 1997; Mosconi, 2005; Yang et al., 2023). Analysis of CSF and brain proteomics indicates that glucose metabolism in astrocytes and microglia exhibits the most notable correlation with AD, compared to other functional networks altered in the disease (Johnson et al., 2020). Moreover, recent studies demonstrate that brain hyperglycemia is positively correlated with the severity of AD pathology and is an attribute of patients with both asymptomatic and symptomatic AD. Hyperglycemia was detected in AD-sensitive brain regions, which also exhibited impaired glycolysis (An et al., 2018). This was the first report of brain hyperglycemia as a feature in AD. The role of brain hyperglycemia and diabetes in AD pathology development remains underexplored. However, recent growing interests in this area are providing more insight. For example, brain hyperglycemia and a high glucose diet were associated with increased Aβ production and plaque deposition, which was mediated by subunit Kir6.2 of the metabolic sensor K_ATP_ channel (Grizzanti et al., 2023). The levels of low-density lipoprotein receptor-related protein-1 (LRP1) – a protein found in the brain endothelium and involved in Aβ clearance into the bloodstream – are reduced in the brain endothelium of diabetic mice. Additionally, hyperglycemic conditions in a blood–brain barrier cell culture model led to lower levels of LRP1 and impaired Aβ40/42 removal (Xue et al., 2022). Furthermore, the use of antihyperglycemic medications in patients with type 2 diabetes reduces the risk of AD and dementia (Torrandell-Haro et al., 2022; Zheng et al., 2023). In light of the increasing associations between glucose metabolic dysfunction and hyperglycemia with AD, it is plausible that glucose buildup in AD brain tissues due to diminished utilization is a contributing factor to AD pathology. This emphasizes the importance of considering the role of hyperglycemia in future studies on AD pathology.

V-ATPase regulation is an intricate process involving numerous players and signaling pathways. Studies, mostly done in non-brain tissues, have attempted to decipher the regulatory mechanisms involved, which, however, remain poorly understood. In our study, the observed decrease in V-ATPase assembly under hyperglycemia may have resulted from multiple mechanisms. Possibilities include alterations to the activity of glycolytic enzymes or their interaction with V-ATPase. This is because V-ATPase is affected by the cellular glycolytic status and exhibits protein–protein interactions with aldolase and phosphofructokinase-1 (Chan & Parra, 2014; Hayek et al., 2019; Lu et al., 2007; Su et al., 2003). Additionally, glycolytic inhibition has been associated with V-ATPase disassembly (Kohio & Adamson, 2013). Future investigation of the interplay between V-ATPase and glycolytic activity under hyperglycemia can be insightful in the context of AD. This is especially important as glycolytic dysfunction is strongly associated with AD pathology (Zhang et al., 2021a; Zhang et al., 2021b) and there are reports of impaired glycolysis in diabetes (Henly et al., 1996; Matsuura et al., 2022; Zhang et al., 2021a). Additionally, acute hyperglycemia can upregulate brain inflammatory mediators (Dorsemans et al., 2017), and inflammatory states are related to mitochondrial and energy deficits (van Horssen et al., 2019). V-ATPase disassembly under glucose starvation has been proposed as a mechanism of energy preservation (Kane, 1995). Thus, if the hyperglycemic conditions used in this study had produced mitochondrial and energy deficits in synaptosomes, then the observed decreased V-ATPase disassembly could have been a mechanism for a similar purpose.

Previous studies have indicated that hyperglycemia increases V-ATPase activity and assembly in mammalian tissues (Kohio & Adamson, 2013; McGuire & Forgac, 2018). The main differences between the prior research and the present study are the duration of hyperglycemia, the tissues analyzed, and the glucose levels used in experiments. Brain tissues were not used in previous studies, in which the glucose levels were more representative of blood glucose levels. Our study analyzed brain tissues with glucose levels representing physiological normo- and hyperglycemic brains. These discrepancies in study designs may contribute to the divergent results.

The V-ATPase activity assay did not show a difference under acute hyperglycemia, which appears inconsistent with the data on V-ATPase assembly. One explanation could be that there are compensatory mechanisms to maintain V-ATPase function in conditions of reduced assembly. To the best of our knowledge, there are no reports of such mechanisms, and the disassembly of V-ATPase is usually reported concomitantly with impaired activity (Hashmi & Kane, 2025; Lu et al., 2008). Another explanation is that the observed reduction of V-ATPase assembly under acute hyperglycemia was modest (mean decrease of 9 %) and insufficient to produce a detectable difference in the amount of released inorganic phosphates. Indeed, several studies that reported a change in V-ATPase activity observed a larger effect on assembly compared to our observation, or a more pronounced impact on assembly than activity. For example, impaired V-ATPase-dependent acidification was reported with a 50 % reduction in assembly (Hashmi & Kane, 2025). Increases in assembly by 90 % and 40 % led only to 17 % and 13 % increases in V-ATPase activity, respectively (McGuire & Forgac, 2018), and a 50 % impairment of glucose-dependent V-ATPase reassembly was reported with a 35 % reduction in V-ATPase-mediated ATP hydrolysis (Chan & Parra, 2014). An additional explanation relates to an inconsistent glucose incubation time in the V-ATPase activity assay due to procedure-specific requirements. In Co-IP experiments, sample incubation was immediately proceeded with pre-clearing and then V-ATPase complex capture, which was performed at 4 °C. In contrast, incubation in the V-ATPase activity assay was followed by ATPase inhibition and then incubation with ATP; these steps were conducted at room temperature for an additional 1.5 h beyond the 10-minute incubation at 37 °C. The necessity of the room temperature steps meant longer sample incubation at a temperature that allows high biological activity compared to 4 °C. This may have led to an effect in V-ATPase activity experiments that is inconsistent with the outcome detected in the ATPase assembly assay. A consistent effect might have been observed if the incubation times were similar. Should this be the case, it could indicate a time-dependent effect of glucose concentration on V-ATPase activity, which might explain discrepancies in the literature regarding the impact of glucose starvation on V-ATPase assembly (Kane, 1995; McGuire & Forgac, 2018; Sautin et al., 2005).

Several limitations to this pilot study need to be acknowledged and further investigated in future research. First, in contrast to acridine orange release, we could not detect a robust response from female mice in the glutamate release experiments. Both experiments assessed synaptic exocytotic activity; however, the measured outcomes differed. The signal detected in glutamate release relied on the physiological levels of glutamate, and it was lower than that obtained with acridine orange. Thus, the signal from female mice might have been insufficient for detection by the experimental *ex vivo* settings in the glutamate release assay. In fact, there are reports of sex differences in the glutamate system that can support this interpretation. For instance, the levels of glutamate in female brains are lower than in male brains in several regions (Al-Suwailem et al., 2018; Frankfurt et al., 1984; O’Gorman et al., 2011). Glutamate has a lower release probability in the nucleus accumbens of female brains (Knouse et al., 2023). Estrogen increases the expression of astrocytic glutamate transporters and glutamate uptake (Pawlak et al., 2005), which is consistent with its neuroprotective effect against glutamate-induced neurotoxicity (Yang et al., 2002). On the other hand, testosterone aggravates glutamate-induced neurotoxicity (Yang et al., 2002), indicating a lower efficiency in buffering extracellular glutamate. Female brains express higher levels of the astrocytic enzyme glutamine synthase, which metabolizes glutamate to glutamine, while they exhibit similar levels of glutaminase, the key enzyme that produces glutamate (Al-Suwailem et al., 2018). Moreover, the affinity of glutamate to certain glutamate transporters and their maximal velocity are higher in synaptosomes from females during specific phases of the estrous cycle (Mitrovic et al., 1999), suggesting more robust glutamate clearance after release. Collectively, these factors predict lower extracellular levels of glutamate in female brains, which is consistent with our inability to detect an endogenous signal in the glutamate release assay.

Second, the model used in this study examined only the effects of acute hyperglycemia *in vitro* on healthy brains. The data are insufficient to infer the impact of chronic hyperglycemia associated with disease states. However, they warrant further exploration of the relationship between hyperglycemia and synaptic vesicular function in animal models of diabetes and AD. This will allow for the investigation of molecular changes over a chronic period of hyperglycemia and provide insight into the impact in an *in vivo* hyperglycemic model. Nonetheless, the present work still presents essential information on how short bursts of hyperglycemia can impact healthy brains, especially since there are reports of acute hyperglycemia rendering brains more vulnerable to insults such as those in ischemia or sepsis (Li et al., 1994, 1995; Warner et al., 1992; Yao et al., 2024). Additionally, acute hyperglycemia can aggravate ischemic brain damage in healthy rats, comparable to the damage observed in diabetic rats (Warner et al., 1992).

A final limitation to consider is the ages of the mice used in the present work. We did not observe significant age-dependent differences for the age range (2–13 months) analyzed in our study. WT mice of this age range are considered young to mid-age healthy adults. It is possible that acute exposure to supraphysiological glucose levels induces similar responses in healthy mouse brains at this adult age range. We speculate that WT mouse brains of older ages may respond differently. Future studies should further investigate the influence of aging and disease status on the impact of chronic hyperglycemic exposure on synaptic vesicular activity and V-ATPase function.

## 5. Conclusions

**Fig. 5.**
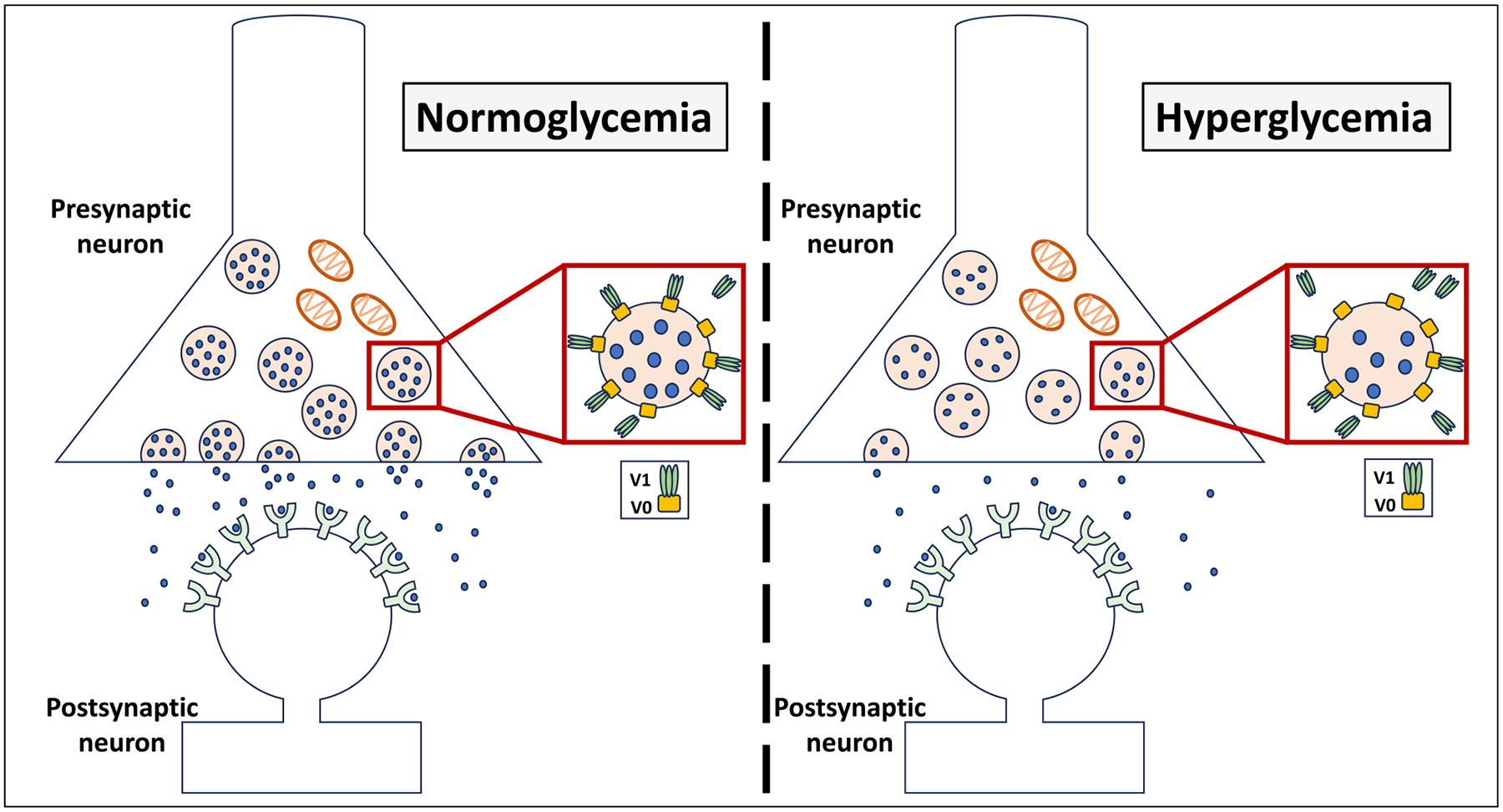
Proposed hypothesis. Acute hyperglycemia reduces synaptic vesicular exocytotic release. Reduced assembly of vesicular V-ATPase could be a potential contributor, considering its essential role in neurotransmitter loading into synaptic vesicles.

The findings in this study provide insight into the possible role of brain hyperglycemia in the development of AD pathology. The results demonstrate that acute exposure to supraphysiological glucose levels, or hyperglycemia, impairs synaptic vesicular release activity and reduces V-ATPase assembly (Fig. 5). Future studies are needed to investigate synaptic vesicular activity and V-ATPase function under chronic hyperglycemic conditions. The mechanisms underlying the hyperglycemic reduction of V-ATPase assembly should also be explored, including the effects of hyperglycemia on the interplay with glycolysis. Furthermore, future studies should also investigate the impact of hyperglycemia on other aspects of AD pathology, for instance, synaptic mitochondrial dysfunction.

## Data Availability

Data will be made available on request.
